# Pain Processing in Cognitive Impairment and Its Association with Executive Function and Memory: Which Neurocognitive Factor Takes the Lead?

**DOI:** 10.3390/brainsci11101319

**Published:** 2021-10-04

**Authors:** Stefan Lautenbacher, Annegret Hoos, Göran Hajak, Wolfgang Trapp, Miriam Kunz

**Affiliations:** 1 Physiological Psychology, University of Bamberg, Markusplatz 3, 96045 Bamberg, Germany; annegret.hoos@googlemail.com (A.H.); miriam.kunz@med.uni-augsburg.de (M.K.); 2 Department of Psychiatry, Sozialstiftung Bamberg, 96049 Bamberg, Germany; goeran.hajak@sozialstiftung-bamberg.de (G.H.); wolfgang.trapp@sozialstiftung-bamberg.de (W.T.); 3 Department of Medical Psychology and Sociology, Medical Faculty, University of Augsburg, 86135 Augsburg, Germany

**Keywords:** neurocognitive, functioning, pain, MCI, executive functioning, memory, cognitive impairment

## Abstract

It is well established that individuals with cognitive impairment present with disturbed forms of pain processing of still unknown origin. As a neurocognitive factor, executive functions have become favored candidates for explanation. For further insights, we aimed at comparing executive functions and memory in their association with parameters indicating onset and escalation of pain perception. Subjective ratings of experimentally induced pressure pain applied in ascending series were assessed in older individuals with (N = 32) and without mild cognitive impairments (MCI) (N = 32). We investigated whether executive functioning (Trail Making Test-B (TMT-B), semantic fluency) or memory (word list and figure recall) were more closely linked to the onset and the escalation of pain. For the MCI group, a strong linkage between pain responses and the TMT-B could be found, i.e., poor test performance was associated with weak pain onset but strong pain escalation. The contribution of memory functions was less substantial and systematic. The prominent role of executive function for pain processing in individuals with MCI could be replicated by a test of cognitive flexibility. This lack of adaptability let individuals with MCI be less vigilant to pain at the beginning but allows for escalating pain in the further course. Thus, being first not sufficiently prepared and later overwhelmed as regards pain may be an early problem in MCI individuals with reduced executive functioning.

## 1. Introduction

There is ample evidence that dementia in its various forms and degrees, affects pain processing [1,2,3,4,5,6]. The majority of findings suggest an enhancement of pain of still unknown origins [1]. From a neurobiological perspective, alterations in prefrontal functioning are of upmost relevance for changes in central nociception and for deteriorating descending inhibitory control [7,8,9]. From a neurocognitive perspective, there is some tentative evidence that worsening of executive functions is the critical mechanism [10,11,12,13], although the impairment of memory is the leading deficit in most forms of dementia [14,15]. This tentative evidence also appears plausible from a theoretical perspective because coping with pain in time requires a readiness of the individual, (i) who has to stop other ongoing activities, (ii) switch attention towards the noxious event, (iii) recall earlier coping attempts, (iv) plan current coping, and (v) finally monitor as well as (vi) evaluate coping and its results. Thus, these components of coping are all subdomains of the overall process conceptualized as executive functions. 

The present study continues to ask which of the two neurocognitive dysfunctions, namely dysexecutive syndrome or memory impairment, shows the strongest linkage to aberrant pain processing in older individuals with cognitive impairments and whether this linkage already exists in the preceding step to dementia, namely mild cognitive impairment (MCI). To properly answer these questions, at least two models of association should be considered. It might be (model 1) that the association between neurocognitive functions and pain processing might be continuous or even linear. Under this perspective, variations in neurocognitive functioning are always linked to variations in pain processing, even at nonpathological levels. There are several studies, which have tried to investigate this by using cross-sectional designs and correlated executive functioning and pain responses in cognitively healthy individuals [11,12,16,17,18,19]. Using such designs, most studies (approximately 80%) did not find significant associations between the two functions [12]. Alternatively (model 2), a threshold of disorder or impairment severity might be considered, postulating that the association of neurocognitive function and pain processing starts only when pathological levels of cognitive impairments are reached. We expect model 2 to be true and thus, investigated the relation between neurocognitive functions and pain processing in two groups of individuals, namely in individuals with no cognitive impairments and in individuals with mild cognitive impairment (MCI). Fitting to the prerequisites for this test, the memory performance [20] as well as executive functions [21] reach already pathological levels of impairment in MCI individuals compared to healthy persons.

Another issue to be considered is that the evidence of an association between dysexecutive symptoms and enhanced pain processing in individuals with cognitive impairments (especially dementia) mainly stems from the use of facial responses as pain indicators [7,10,22]. Although facial responses to pain have been shown to be valid pain indicators [23,24], they are expressive behaviors which are regulated by inhibitory mechanisms [25,26] and may be as such sensitive to executive functions. Under this circumstance, executive function may influence especially the facial display of pain without directly impacting the processing of pain. Therefore, the notion of an association between executive functioning and pain processing in cognitively impaired individuals would be strengthened if it can be replicated, this time with subjective self-reported data as the gold standard of pain assessment. Such a replication is of course only possible in a sample of individuals where the cognitive impairment is only mild so that a valid self-report of pain is still possible. The ability to provide valid pain reports makes it possible to investigate psychophysical curves across ascending series of pain intensities, which allow for estimating the early onset (intercept) and the later escalation (slope) of pain perception and, by that, to learn more about the stages of pain and their corresponding neurocognitive influences, which has yet not been investigated in individuals with MCI. 

Thus, we aimed at investigating the association between executive function and memory on the one hand and pain processing on the other hand to compare the explanatory power of the two neurocognitive functions in two groups of individuals, namely (i) in individuals with MCI and (ii) in healthy controls (HC) without cognitive impairments. We assumed an association only when the individuals reached pathological levels of neurocognition (MCI group). We intentionally selected participants who still could provide a self-report to the experimentally induced pain, suffering therefore only from MCI. Our hypothesis was that executive dysfunctions explain pain perception (intercept and slope of the psychophysical curve) better than memory impairment in individuals with cognitive impairment, whereas no relationship between cognitive performance and pain responses can be found in cognitively healthy individuals. 

## 2. Materials and Methods

### 2.1. Participants 

In total, 74 individuals over the age of 65 years (following the WHO definition of older individuals we recruited individuals over the age of 65) were recruited for the study via local newspaper advertisements and amongst inpatients and outpatients from the Sozialstiftung Bamberg, Clinic for Psychiatry, Psychosomatic Medicine and Psychotherapy. Exclusion criteria in the present study were: participants suffering from severe forms of dementia (Mental State Examination test [27]; MMSE < 17), peripheral and central neuropathy, Parkinson’s disease, strokes with neglect, aphasia or paresis, and psychiatric disorders due to their known impact on pain. 

For the group of individuals with MCI, inclusion criteria were (a) subjective memory complaints, (b) impairment in at least one cognitive domain (a score of one standard deviation below the mean score of the control group within one of the tests of the CERAD-Plus), and (c) a score of ≤ 28 on the Mini Mental State Examination test (MMSE [27] based on previously published cutoff scores [28]). We used the Consortium to Establish a Registry for Alzheimer’s neuropsychologic battery (CERAD-Plus [29] to apply an established battery of multiple neuropsychological functions. Based on the above listed criteria, 32 participants were classified as MCI and 42 as cognitively healthy controls (HC). To keep the sample size equal between groups and to have comparable gender- and age-distribution in both groups, we had to exclude the 10 youngest females from the group of HC. The demographic data of all N = 64 participants who were entered into analyses can be found in Table 1.

The study was approved by the ethics committee of the medical faculty of the Friedrich-Alexander-University Erlangen-Nürnberg (number: 168_12 B). All subjects still had legal capacity and a written informed consent was obtained from all participants. We took care that information and instructions were given in a simple fashion adequate to individual cognitive capacities. All subjects received monetary compensation for their participation. 

### 2.2. Materials and Procedure 

The study was composed of two blocks. In a first block, neuropsychological functioning was assessed. In the second block, the experimental pain testing took place. 

Block 1—neuropsychological testing: In the first block (45 min), a short screening questionnaire was carried out to assess demographic information and to again check for exclusion criteria (these were already assessed during the recruitment). Thereafter, the participant’s cognitive performance was assessed using the CERAD-Plus [29]. In its current version, the CERAD-Plus battery includes the Mini-Mental State Examination [27], as well as 11 other tasks including phonematic fluency, semantic fluency, naming (Boston Naming Test), word list learning, delayed free recall and recognition of a word list, figure drawing (copying geometric shapes), delayed figure recall, and the Trail Making Tests, forms A and B. For the present study, we focused on two areas of cognitive functioning, namely executive functioning and memory performance.

Memory domain: Based on previous classifications [30], the memory domain comprises “word list recall” (recalling a list of 10 words 20–30 min later) and “figure recall” (redrawing four geometric figures 20–30 min later). 

Executive domain: Based on previous classifications [30], the executive domain included the “Trail Making Test Part B” (TMT-B) (connecting numbers and letters in a sequential alternating order), which is a measure of cognitive flexibility. Since the test performance is also based on other cognitive factors (e.g., selective attention and processing speed), the flexibility proportion is extracted by computing the ratio between performance in part B divided by A (only numbers must be connected in part A) as proposed by Salthouse [31]. We therefore computed the TMT-B/A score. Moreover, “semantic fluency” (number of animal names generated within 60 s.) was taken as second indicator of executive functioning. The domain of executive functioning is very broad and in the present study we only included two tests of executive functioning that are well established for neuropsychological testing in individuals with MCI and dementia and are an integral part of the CERAD test battery. However, other aspects of executive functioning (e.g., updating and inhibition) were omitted in the present study. 

Block 2—experimental pressure pain: In the second block (20 min) experimental pressure pain was administered. Experimental pain was induced by using pressure stimuli of four ascending intensities (50, 200, 400, and 500 kPa) applied with a hand-held pressure algometer (Algometer Type II, SOMEDIC Electronics, surface 1 cm^2^). The algometer has a built-in pressure transducer, an electronic recorder, and a display unit to monitor the applied pressure as well as the rate of rise. Following a previous protocol [3,7] pressure was applied to the shoulder (upper mid part of the right and left trapezius muscle halfway between the neck line and the shoulder line) and to the forearm (midportion of the right and left inner forearm, halfway between wrist and crook of the arm, arm positioned stable on a table). In total 16 pressure stimuli were applied (4 intensities × 2 body side × 2 body sites). Starting point was the shoulder of the dominant body side, followed by the shoulder on the nondominant side. After this, the same was repeated on the dominant and nondominant forearm. Pressure was increased steadily within two seconds until maximum stimulus intensity was reached and then continued at that level for another five seconds. Right after each stimulation, participants were asked to provide a self-report rating about the peak sensation felt. The self-report was assessed via a 5-point verbal category scale (no pain (1)–mild pain (2)–moderate pain (3)–strong pain (4)–very strong pain (5)). For later analyses, ratings were averaged across body sides. The interstimulus interval varied between 20 and 30 s. In order to familiarize subjects with pressure stimulation and the rating procedure, three stimuli of 200, 50, and 400 kPa were applied in this order to the thigh before testing started. For later analyses, we computed regression lines to capture the stimulus-response relationship for each participant across the ascending order of pressure pain intensities. These relationships between stimulus and response intensities can be described in terms of their onset (intercept) and their rate of increase (slope). For each participant, intercept and slope were calculated separately for the two stimulation sites (shoulder, forearm). 

After the experimental pain block, participants were asked to view and evaluate a set of emotional pictures (happy, disgust, or neutral). These data were not part of the present study.

### 2.3. Statistical Analysis

Group differences in executive functioning and memory: to investigate whether groups (HC vs. MCI) differed in their cognitive performance, raw scores of the four tests (“TMT-B/A” and “semantic fluency”; “word list recall” and “figure recall”) were compared between groups using multivariate analysis of variance. 

Group differences in pain ratings: to investigate whether groups (HC vs. MCI) differed in their ratings of the pressure stimuli, analyses of variance with repeated measurements (within-subject factor: “pressure intensity” and between-subject factor: “group”) were conducted separately for the two body sites (“shoulder” and “forearm”). 

Executive functioning and memory performance predicting pain responsiveness: With regard to the main aim of the present study, namely, to test whether executive functioning or memory are more closely linked to pain rating, hierarchical regression analyses were computed. The executive function and memory performances were entered as predictors. In line with the theoretical background, executive functions were first entered into the model. In the next step, memory functions were added to the previous model to see whether the newly added variables could account for a significant increase in explained variance in the dependent variables. As dependent variable, we entered “intercept” and “slope” values, respectively, as indicators of the early onset (intercept) and the later escalation (slope) of pain responsiveness. Analyses were conducted separately for both groups (HC and MCI), for both stimulation sites (shoulder and forearm) and for both psychophysical parameters (intercept and slope), thus resulting in eight regression analyses.

Data were analyzed with the IBM SPSS statistics software (version 25, IBM, New York, USA). Two-tailed *p*-values are reported, and *p* < 0.05 was considered significant.

## 3. Results

Table 1 presents the demographic data of the participants. As can be seen, the two groups were comparable in sample size as well as with regard to educational years and gender- and age-distribution. Nevertheless, given that the age comparison between groups just missed the level of significance, we wanted to ensure that our findings were not confounded by age. Therefore, we correlated age with our pain outcomes and found no significant associations (all *p*-values ranging between 0.33 and 0.56).

Group differences in executive functioning and memory: As expected, groups differed significantly in their cognitive performance (F(4,59) = 7.21; *p* < 0.001). As univariate outcomes showed, HC and MCI differed in all tests: executive functioning (TMT-A/B: F(1,62) = −4.20, *p* = 0.045; semantic fluency F(1,62) = 6.11, *p* = 0.016) and memory performance (word list recall: F(1,62) = −26.20, *p* < 0.001; figure recall: F(1,62) = 5.08, *p* = 0.028) with MCI individuals showing lower cognitive functioning in all four tests (see Table 1). 

**Figure 1:**
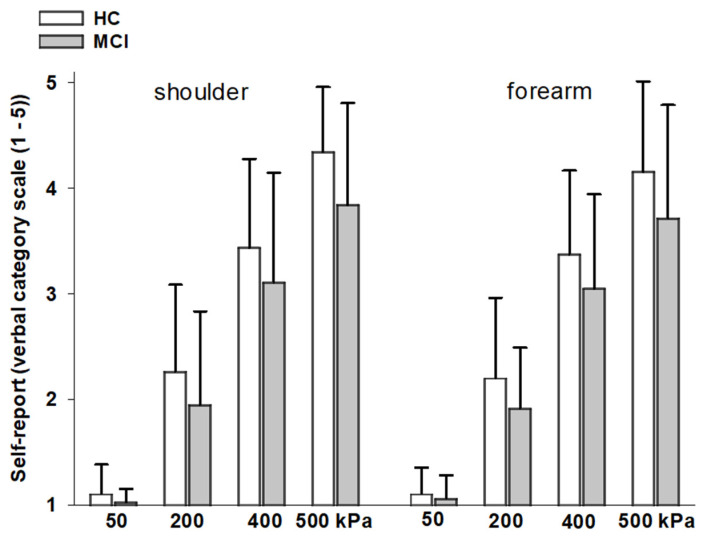
Pain responses (verbal ratings, mean and SD) across pressure intensities. Values are given separately for the two stimulation sites and separately for the two groups.

Group differences in pain ratings: As can be seen in Figure 1, pain ratings significantly increased across pressure intensities, both at the shoulder (F(3,186) = 447.28, *p* < 0.001) and the forearm (F(3,186) = 452.27, *p* < 0.001). This increase did not differ between groups, as indicated by nonsignificant interaction effects between “group” and “pressure intensity” (shoulder: F(3,186) = 1.92, *p* = 0.129; forearm: F(3,186) = 1.99, *p* = 0.116). There were also no main group effects on pain ratings (shoulder: F(1,62) = 3.80, *p* = 0.056; forearm: F(1,62) = 3.07, *p* = 0.085).

In line with this, intercept and slope values (derived from the individual’s psychophysical curve of pain ratings across pressure intensities) also did not differ between groups (F(4,58) = 0.744, *p* = 0.566; see also Table 1).

### Executive Functioning and Memory Performance Predicting Pain Responsiveness

The results of the regression analyses can be found in Table 2 (slope) and Table 3 (intercept), respectively.

HC: As can be seen on the left side of Table 2 and Table 3, in HC, cognitive performances could not significantly predict the increase in pain ratings across pressure stimuli (slope), or the onset (intercept). Indeed, neither did we find significant prediction when entering the executive functioning in the first step nor when also entering memory performance in the second step of the regression model.

**Figure 2:**
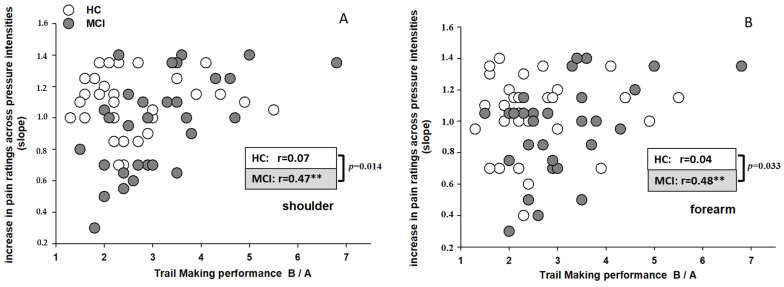
Associations between the escalation in pain perception across pressure stimuli (slope) and executive functioning (TMT-B/A) for the two stimulation sites (**A**). shoulder; (**B**). forearm). The different groups (HC, MCI) are displayed in different shades of grey. Partial correlation coefficients (r-values) are displayed separately for each group. Significant r-values are specified by ** (*p* < 0.01). The *p*-values refer to the comparison of correlation coefficients between groups using z-statistics.

MCI: In contrast, for the group of MCI we did find significant associations. As can be seen on the right side of Table 2, entering executive functioning in the first step led to a significant prediction of the escalation (slope) of pain responses across pressure intensities, with executive functioning explaining 23% (stimulation site: shoulder) and 24% (stimulation site: forearm) of the variance in pain escalation, respectively. As can be seen in Table 3, it was especially the performance in the TMT-B/A that showed a significant prediction of pain escalation. Entering memory performance in the second step, did not lead to a significant increase in explained variance. To better visualize this association between TMT-B/A performance and pain escalation (slope), these values are displayed as scatterplots in Figure 2A (shoulder) and Figure 2B (forearm). The different groups (HC and MCI) are displayed in different shades of grey. As can be seen in these Figures, the poorer the executive functioning (indicated in higher TMT-B/A scores) the steeper is the slope in pain ratings (indicated by increasing values) within the MCI group. For the HC group, no such associations can be seen. When comparing the r-values displayed in Figure 2 between groups using z-statistics, we found that the correlations differed significantly between HC and MCI.

**Figure 3:**
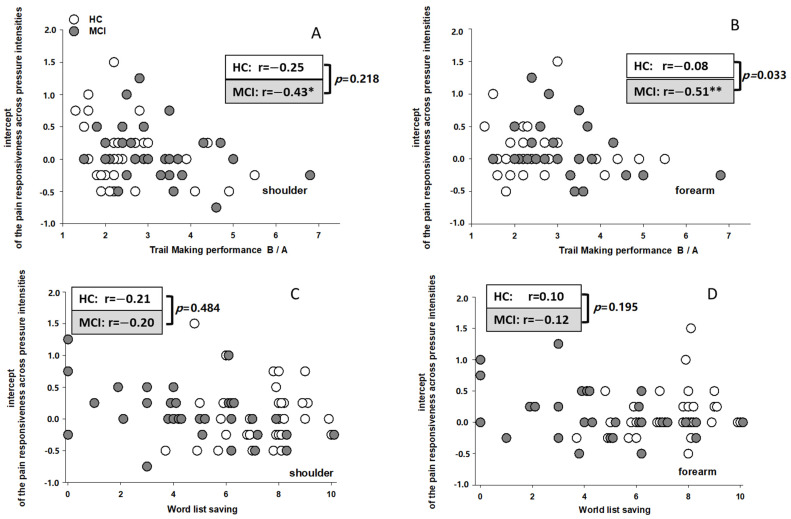
Associations between the onset of pain perception (intercept) and executive functioning (TMT-B/A) (top row) and memory function (word list saving) (bottom row) for the two stimulation sites ((**A**,**C**) shoulder; (**B**,**D**) forearm). The different groups (HC, MCI) are displayed in different shades of grey. Partial correlation coefficients (r-values) are displayed separately for each group. Significant r-values are specified by * (*p* < 0.05) and ** (*p* < 0.01). The *p*-values refer to the comparison of correlation coefficients between groups using z-statistics.

With regard to the onset (intercept) of pain perception, we again found significant associations with cognitive performances in the group of MCI. As can be seen on the right side of Table 3, entering executive functioning in the first step led to a significant prediction of the intercept values when stimuli were applied to the forearm, with executive functioning explaining 23% of the variance. Entering memory performance in a second step led to a further significant increase in explained variance, with memory explaining an additional 28% (shoulder) and 20% (forearm) of the variance in intercept values. As can be seen in Table 3, it was especially the performance in the TMT-B/A and in the word list saving test that allowed a significant prediction of pain onset. To better visualize these associations between neurocognitive functions, i.e., TMT-B/A and memory performance, and pain onset (intercept), these values are displayed as scatterplots in Figure 3A,B (TMT-B/A) and 3c/d (word list saving test). The different groups (HC and MCI) are displayed in different shades of grey. As can be seen in Figure 3A,B, the poorer the executive functioning (indicated in higher TMT-B/A scores) the lower the intercept values within the MCI group. Moreover, as can be seen in Figure 3C,D, the better the memory performance (more words being recalled) the lower the intercept values within the MCI group. For the HC group, no such associations can be seen. When comparing r-values between groups using z-statistics, we found that only the correlation between TMT-B/A and intercept values for pain being applied to the arm (see Figure 3B) differed significantly between HC and MCI. For the other associations between cognitive functioning and the onset of pain perception, no significant group differences were found (see Figure 3A,C,D).

## 4. Discussion

The major aim of the present study was to demonstrate the relative explanatory power of executive function and memory for predicting subjective responses to pain in individuals with MCI and in cognitively healthy controls (HC). There was clear evidence that cognitive flexibility (TMT-B/A), as a subdomain of executive functions, appeared associated with a weak response at pain onset (intercept of the linear psychophysical function between stimulus intensity and ratings) and a late strong escalation of pain perception (slope of the linear psychophysical function between stimulus intensity and ratings) across ascending series of pain stimuli. Interestingly, these associations emerged only in individuals with MCI, whereas no associations were found in HC. Despite these differences in associations between groups, the pain responses themselves did not differ between the two groups. With regard to memory performance and its association with pain responses, we found some but less substantial and systematic influence on pain processing compared to executive functioning. Thus, we could again demonstrate the influence of executive functions (cognitive flexibility, (TMT-B/A)) on pain processing in individuals with cognitive impairments [10]. In MCI, individuals with poorer executive functioning seemed less prepared to detect weak pain at the beginning and showed an escalating pain response at further increases of pain intensity. It may well be that cognitive inflexibility lowered the adaptability to the situational demands in pain processing, and by that, the first signals of pain could not be used to activate pain coping or pain inhibition at later stages of pain processing. This means that MCI individuals and even more people with dementia are often not sufficiently readied for pain even if the warning signals for impending pain are clear for cognitively healthy individuals. The consequence likely is that self-initiated attempts of pain coping rarely occur. In painful medical procedures, the clinical staff should be aware of this handicap and present other analgesic protection (analgesics and distraction of attention). 

As we assumed, this association between cognitive inflexibility and aberrant pain processing occurred only in individuals with MCI and thus, a certain pathological level of cognitive impairment seems necessary to reveal such association. One might argue that similar associations might be found also in cognitively healthy individuals when using tools allowing more fine-grained assessment of executive functions and that we missed such associations by using tests targeted at individuals with dementia (i.e., CERAD). However, previous evidence has also pointed into weak/inconsistent [12] or even no associations between self-report of pain and executive functioning in cognitively healthy individuals, even when using neuropsychological tests of executive functioning developed for cognitively healthy individuals [17].

Although the neurocognitive influences on pain processing differed between HC and MCI, interestingly, the pain responses themselves (including intercept and slope values across pressure intensities) did not differ between groups. Thus, we could not replicate an increased pain responsiveness as observed in patients with dementia [2,3,4,7]. However, we already found in an earlier study that individuals with MCI show comparable pain responses (pain ratings, RIII reflex, facial responses, sympathetic skin response, and evoked heart rate response) to age-matched individuals without cognitive impairments [32]. Similar findings were reported for early stages of dementia (e.g., [5]). Thus, it may be that the first changes regarding the mechanisms of pain processing may become already manifest in the early stages of cognitive impairment (MCI), however, these changes might be too small to already manifest themselves in detectable changes in pain responses. 

According to our present and earlier data, we put forward the following model of changes across the course of cognitive impairment (from MCI to dementia). At the initial development of cognitive impairments, the loss in executive functioning is accompanied with a loss in the adaptability to new situational demands, and by that, individuals become less vigilant to pain. In consequence, they may miss the first indications of pain and do not sufficiently prepare to cope with and adapt to increasingly stronger pain levels. Therefore, pain may escalate and MCI persons with low executive functioning might become overwhelmed. In more advanced stages of cognitive impairment (moderate stages of dementia), the persons can no longer remember these failures and have to re-experience this cycle of ineffective pain coping and inhibition again and again without any ability to improve executive functions in this respect. At this point, noxious events at all intensities repeatedly lead to strong pain responses [2,3,4,7]. Future studies should test this developmental model at best in longitudinal studies. 

As expected, we found clear associations of executive function, namely cognitive flexibility, with pain processing in MCI individuals partly for the onset (intercept) and especially for the escalation (slope) of pain across pressure intensities. As expected, the associations between memory performance and pain responses were less consistent and it was only the onset (intercept) of pain that showed associations with the performance in the word recall test in individuals with MCI. Before interpreting this association, we have to admit that the linear psychophysical curve we computed to gain intercept and slope values was based on only few data points (four pressure intensities) and thus, is not always reliable and such attempts may lead to chance findings. So, what does it mean that a low word recall performance in MCI is associated with high intercept scores for pain responses? One may speculate that not remembering and foreseeing what will soon happen led the individuals to be already startled when only touched by the stimulator (thus, over-rating the low-pressure intensities). Unfortunately, our data do not allow more than these speculations. Interestingly, however, our finding of a differential association between memory compared to executive functioning and pain (a low intercept score was associated with low executive but high memory function) is in line with previous findings. Madariaga et al. [13] also found reverse effects of memory and executive functioning on clinical pain in older individuals with various degrees of cognitive impairments. Whereas poor executive functioning correlated with higher pain scores (similar to our slope findings), poor memory functioning correlated with lower pain scores. Thus, our findings corroborate the notion that executive functioning and memory performance are independently associated with pain responses in older individuals with cognitive impairments.

The present study provides evidence that the assessment of self-report of pain and not only the assessment of facial pain responses allows for the conclusion that executive functioning is associated with pain responses in individuals with cognitive impairments. However, future studies should attempt to assess verbal and nonverbal pain indicators in parallel. Furthermore, although the association between pain responses and executive functions in individuals with cognitive impairments (MCI and dementia) has appeared to be robust; there is some variability with regard to which subcategory of executive functioning plays the most important role. Abstract thinking, making plausibility judgments [10], cognitive inhibition, shifting [19], as well as cognitive flexibility (the present study) have been suggested as most important predictors for pain responses in MCI and dementia. Thus, a taxonomy is needed to differentiate between those executive functions that influence pain response and those not sharing this functional property. Lastly, clinical attempts to find associations between dysexecutive syndromes and chronic pain in individuals with cognitive impairments, which have already begun [33], should be continued [13] and integrated with our laboratory approaches. 

## 5. Conclusions

In conclusion, we found that cognitive flexibility proved to be consistently associated with pain only in individuals with MCI; low flexibility was associated with little pain at onset and with notable pain escalation across increasing pain intensities. As clinical implication, our findings suggest that better executive functioning might be a protective factor against pain. In this sense, neurocognitive remediation used in the treatment of MCI and dementia may also become a tool in pain management for these patients.

## Figures and Tables

**Table 1:** Demographic data of the sample studied.

		HC	MCI	Group Differences (*p*-Values)
N		32	32	1.000
Age (in years); mean (SD)		71.6 (3.4)	73.7 (5.5)	0.067
Sex ratio (female/male)		17/15	10/22	0.128
Education (years); mean (SD)		12.3 (3.6)	11.2 (3.2)	0.227
**Neuropsychological functioning**				
MMSE; mean (SD)		29.6 (0.5)	25.9 (2.7)	**<0.001**
Executive functioning; mean (SD)	Semantic fluency	22.6 (6.9)	18.9 (5.4)	**0.016**
	TMT-B/A	2.6 (1.0)	3.1 (1.1)	**0.045**
Memory functioning; mean (SD)	Word list recall	7.4 (1.5)	4.8 (2.5)	**<0.001**
	Figure recall	8.9 (2.5)	7.3 (3.1)	**0.028**
**Onset (intercept) and escalation (slope) of the psychophysical curve across pressure intensities**				
slope-shoulder		1.1 (0.2)	1.0 (0.3)	0.138
slope-forearm		1.0 (0.3)	0.9 (0.3)	0.292
intercept-shoulder		0.07 (0.49)	0.06 (0.44)	0.934
intercept-forearm		0.13 (0.39)	0.14 (0.40)	0.960

Note. SD: standard deviation, TMT: Trail making test; HC: healthy controls; MCI: mild cognitive impairment, significant group differences are marked in bold.

**Table 2:** Hierarchical regression analysis for executive and memory functions predicting the slope of pain responsiveness (increase in pain ratings across all pressure intensities). Findings are given separately for the two stimulation sites and the two subject groups.

	Steps	Predictor	HC					MCI				
			β	T	R^2^	ΔR^2^	*p*(ΔR^2^)	β	T	R^2^	ΔR^2^	*p*(ΔR^2^)
**Stimulation site: shoulder**	1				0.01	0.01	0.904			0.23	0.23	0.028 *
		Semantic fluency	0.01	0.43				−0.01	−0.10			
		Trail making B/A	0.01	0.05				0.13	2.86 **			
	2				0.13	0.12	0.182			0.30	0.06	0.344
		Semantic fluency	0.01	1.10				−0.01	−0.30			
		Trail making B/A	−0.01	−0.35				0.14	2.87 **			
		Word list savings	−0.01	−0.42				0.04	1.49			
		Figure savings	−0.02	−1.52				−0.02	−0.66			
**Stimulation site: arm**	1				0.02	0.02	0.705			0.24	0.24	0.031 *
		Semantic fluency	−0.01	−0.67				0.01	1.65			
		Trail making B/A	0.03	0.60				0.11	2.41 *			
	2				0.15	0.13	0.147			0.34	0.10	0.193
		Semantic fluency	0.00	−0.04				0.01	1.01			
		Trail making B/A	0.01	0.18				0.12	2.62 *			
		Word list savings	0.02	0.47				0.04	1.88			
		Figure savings	−0.04	−1.99				−0.01	−0.68			

Note. N = 64; * *p* < 0.05, ** *p* < 0.01; HC = healthy controls; MCI = mild cognitive impairment.

**Table 3:** Hierarchical regression analysis for executive and memory functions predicting the onset of pain perception (intercept). Findings are given separately for the two stimulation sites and the two subject groups.

	Steps	Predictor	HC					MCI				
			β	T	R^2^	ΔR^2^	*p*(ΔR^2^)	β	T	R^2^	ΔR^2^	*p*(ΔR^2^)
**Stimulation site: shoulder**	1				0.12	0.12	0.147			0.07	0.07	0.399
		Semantic fluency	−0.01	−1.10				−0.01	−0.50			
		Trail making B/A	−0.13	−1.51				−0.10	−1.29			
	2				0.13	0.01	0.935			0.35	0.28	0.012 *
		Semantic fluency	−0.02	−1.12				0.02	1.11			
		Trail making B/A	−0.12	−1.36				−0.15	−2.23 *			
		Word list savings	0.01	0.19				−0.10	−2.70 *			
		Figure savings	0.01	0.21				−0.02	−0.62			
**Stimulation site: arm**	1				0.05	0.05	0.477			0.23	0.23	0.036 *
		Semantic fluency	0.01	1.09				−0.03	−1.92			
		Trail making B/A	−0.05	−0.73				−0.13	−2.12 *			
	2				0.11	0.06	0.431			0.43	0.20	0.031 *
		Semantic fluency	0.01	0.53				−0.01	−0.60			
		Trail making B/A	−0.03	−0.44				−0.17	−2.81 *			
		Word list savings	0.02	0.40				−0.07	−2.39 *			
		Figure savings	0.03	0.98				−0.01	−0.47			

**Note.** N = 64; * *p* < 0.05; HC = healthy controls; MCI = mild cognitive impairment.

## Data Availability

The descriptive data of the study can be provided by contacting the corresponding author of the study.
